# The Costs and Underappreciated Consequences of Research Misconduct: A Case Study

**DOI:** 10.1371/journal.pmed.1000318

**Published:** 2010-08-17

**Authors:** Arthur M. Michalek, Alan D. Hutson, Camille P. Wicher, Donald L. Trump

**Affiliations:** Roswell Park Cancer Institute, Buffalo, New York, United States of America

## Abstract

Arthur Michalek and colleagues explore the consequences of scientific misconduct and, using a single case as an illustration, estimate that direct costs can approach US$525,000, although total costs are likely to be higher.

**Summary Points**

- The consequences of scientific misconduct are far-ranging and the costs associated with their investigation are substantial.
- It is possible to estimate the cost (direct and indirect) of investigating a single case of scientific misconduct.
- For a specific investigation for which costs were estimated for all phases of the review process, direct cost estimates approached US$525,000.
- For an individual country, the total costs to associated with the review of all cases of scientific misconduct, both reported and not reported to the Office of Research Integrity, are likely to be exponentially higher.

Fallout from scientific misconduct can be pervasive. From the broadest perspective, the public, current and future patients, funding agencies, and even the course of research may be adversely affected by scientific misconduct. At the local level. members of the perpetrator's laboratory, colleagues, trainees, and the financial resources and reputation of the home institution may become tainted. The costs associated with these acts are substantial. This article will present a model we have developed to estimate the monetary costs of scientific misconduct. Estimates are based on a case that occurred at our institution, the Roswell Park Cancer Institute, which is a National Cancer Institute–designated Comprehensive Cancer Center located in the United States. Our experiences will likely not be wholly representative of other institutions, but we feel could be instructional and should serve as a guide in the calculation of costs at other institutions.

Scientific misconduct is defined by the US Office of Research Integrity (ORI) as “fabrication, falsification, or plagiarism in proposing, performing, or reviewing research or in reporting research results” [1]. The misconduct must be “committed intentionally, knowingly, or recklessly, and there must be a significant departure from accepted practices” [1].

Scientific misconduct likely dates back to the earliest days of scientific inquiry. Fanelli [2] conducted a meta-analysis of published surveys that asked scientists whether they or a colleague had ever committed scientific misconduct. Approximately 2% of respondents admitted to have committed scientific misconduct and 14% reported knowledge of such behavior by their colleagues [2]. The deleterious effects of these transgressions on the scientific knowledge base cannot be overstated. A poignant example is related by Shafer in his review of Scott Reuben's fraudulent research, which comprised 21 articles and abstracts spanning 15 years [3]. These articles focused on the long-term beneficial effects of perioperative nonsteroidal anti-inflammatory drug administration. As Shafer so eloquently stated, this misinformation “is deeply woven into many review articles, meta-analyses, lectures summaries, and the memories …” of individuals exposed to this information. The obvious questions are: can we re-educate everyone who has been swayed, consciously or unconsciously, by fraudulent research and, if so, how?

## Assessing the Costs of Scientific Misconduct

The costs associated with scientific misconduct can be divided into three domains: conduct of the fraudulent research, investigation, and remediation.

### Costs Associated with the Conduct of the Fraudulent Research

These costs includes all monetary investments (institute start-up funds, grant funding) made in the fabricated research as well as intangibles such as loss of productivity of the associated research group, loss of trust, the demoralization of faculty/trainees, and misdirection of the research efforts of other labs. In some cases, the institution may be required to reimburse the funding agency for costs of the fraudulent research as well as pay penalties, and in certain instances, temporarily suspend other studies during the investigation.

### Investigative Costs

An aspect frequently overlooked in the discussion of misconduct costs are those directly related to the investigation. These costs vary considerably and are dependent on the nature of the incident (type of misconduct, complexity, etc.) and the associated time required to investigate. However, all investigations share similar elements that need to be considered when calculating overall costs. At our institution, in keeping with the model proposed by the ORI, allegations of misconduct proceed through three levels of review, each assuming escalating responsibilities and costs.

At our institution, allegations are initially reviewed by the Vice President for Corporate Ethics and the Dean of Educational Affairs. If the allegation is determined to have merit, an inquiry is initiated. This second level requires review by a committee appointed by the Vice President for Corporate Ethics and the Dean. Membership consists of the Vice President for Corporate Ethics, the Dean, four faculty members, and an attorney. The Inquiry Committee determines whether there is sufficient evidence of possible research misconduct to warrant an investigation. The inquiry is not intended to reach a final conclusion about whether research misconduct definitely occurred or who was responsible. That is the role of the Investigation Committee, which is appointed by the Vice President for Corporate Ethics and the Dean. Membership is broader and includes other professional expertise. Membership consists of the Vice President for Corporate Ethics, the Dean, at least two individuals from outside the unit or department of the complainant(s) who are expert in the subject matter or scientific area, a statistician, a representative from Human Resources, an attorney, and any other members deemed appropriate. The purpose of the investigation is to explore the allegations in detail, to examine the evidence in depth, and to determine specifically whether research misconduct has been committed, by whom, and to what extent.

Costs of the investigation may be divided into personnel (committee membership, witnesses, and support staff), material costs, and consultant costs. The most expensive component of any investigation is faculty time. Faculty members engaged in our reviews are usually associate or full professors. Faculty members on investigation committees spend considerable time both in and out of the formal committee meetings. Time spent outside formal meetings is directed at reviewing materials, securing additional information, reanalysis of data, writing, and other preparatory activities. Our experience is that faculty spend anywhere from three to ten times more time working outside of meetings than they do in meetings. Individual time commitments vary based on the individual's expertise as well as committee assignments. Costs associated with witnesses' time must also be considered. The number and frequency of witness interviews varies based on the complexity of the investigation. Witnesses also spend time outside of meetings preparing their testimony. Administrative support costs include secretarial and clerical time needed for transcription of recordings, photocopying, filing, and other related tasks. Most investigations will require sequestration of physical materials including all laboratory notebooks, computers, and other electronic storage devices. At times forensic computer experts are required to analyze hard drives as well as to retrieve e-mail exchanges or other documents that are still resident on the institutional server.

### Remediation Costs

These costs include those necessitated by program closure. Not only are funds previously invested in the fraudulent research lost, but so too are funds currently supporting the fraudulent research. Moreover, pending grant applications may be recalled and further funding of existing grants may be delayed or lost. Loss of funding can be devastating to the honest members of the affected laboratory. A myriad of administrative decisions need to be made regarding such things as continuance of trainees (pre- and postdoctoral) and staff members from the affected lab, impact on trainee's research; and the costs of possibly phasing out bona fide research conducted by the guilty party. Other less-obvious costs include reputational damage to the institution, which may affect competitiveness of future grants as well as fundraising and, for those involved in patient care, there is potential patient harm and loss of patient trust and revenue. Institutional expenses may also include the cost of civil legal action from patients. Extrainstitutional costs may include intellectual corruption of the scientific literature, misdirection of future research, costs to journals in retracting deceptive research, and costs to revise guidelines based on fraudulent research.

## A Possible Statistical Approach for Scientific Fraud Analyses

Very little research has been done to develop methods for formally modeling the cost of scientific fraud. Research in this area has been directed primarily at attempting to model the behavior of the individual scientist with respect to incentives for committing a fraudulent act. It has been our aim to develop a data-based modeling approach aimed at better understanding the factors that contribute to the overall cost of scientific fraud.

We are aware of no published methodological research with respect to modeling the factors that contribute to an estimate of the average aggregate cost (AC) of fraud. The purpose of such models would be two-fold: (1) Identifying the most significant factors associated with the cost of the “average” misconduct case in terms of the proportion of variation explained and (2) prediction of the aggregate cost of a misconduct case conditional on measureable factors. A semi-additive model of AC might take the form
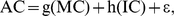
where g and h represent known functional forms, e.g., a linear model, and

- MC = measurable costs
- IC = intangible costs
- ε = stochastic error.

Examples of some ICs would include loss of future earnings related to a line of research; reputational damage to the institution, which may affect competitiveness for future grants and contracts; negative effects on fundraising; and, for those involved in patient care, loss of patient revenue.

Practically speaking, a more manageable working model would take the form
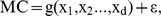
where x_1_, x_2_, …, x_d_ represent the d factors for which a given institution or institutions have the ability to measure and collect in an administrative database and ε again represents stochastic error. Based on our experiences at Roswell Park Cancer Institute, a list of factors to be considered for inclusion in a model of cost include, but is not limited to the following:

- x_1_ = grant direct and indirect dollars returned to the funding agency,
- x_2_ = institutional legal costs,
- x_3_ = hourly cost of faculty time commitment to an investigation panel,
- x_4_ = cost of sequestration of evidentiary materials,
- x_5_ = human resource–related costs,
- x_6_ = institutional start-up costs for supporting the fraudulent research,
- x_7_ = Institutional Review Board–related costs for suspending and closing clinical studies,
- x_8_ = Institute Animal Care and Use Committee–related costs for suspending and closing animal studies,
- x_9_ = payment of penalties related to tainted research,
- x_10_ = hourly costs associated with retracting published research,
- x_11_ = hourly costs of specialized consultants needed for advisement to the investigation panel.

To date we have not gathered cost factor information prospectively or with any degree of precision in order to fit these types of models. Hence, our cost estimates to date amount to a “best guess” scenario, as illustrated in the next section. Ultimately, to apply this model a database will be developed from which we can examine statistically the relative contributions of each factor to the MC. Then, for example, the fitted model then may be utilized for estimating the cost of a future misconduct case in terms of resource management.

## Applying This Approach to a Case

The following case study was based on an actual investigation. Cost estimates are given in US dollars.

### 

#### Allegation

An allegation of research misconduct was made against a senior scientist for enhancing and fabricating images and data contained in a federal grant application.

#### Action

The allegation, in accordance with institute policy, was reviewed by the Vice President for Corporate Ethics and the Dean. A determination was made that there was sufficient credible and specific potential evidence of research misconduct to warrant an inquiry. The deliberation and data gathering to support this decision cost approximately $1,000.00.

#### Inquiry

An Inquiry Panel was convened consisting of the aforementioned membership. The Panel reviewed the grant application in question, additional information regarding more than a dozen figures in the grant, as well as e-mail correspondence between the respondent and several staff members. The panel concluded that there was sufficient evidence to support the allegation and that an investigation was warranted. Panel time required to review and discuss data to support this decision cost about $13,000.00.

#### Action

At this point the respondent's laboratory equipment was sequestered as were all lab notebooks, computer hard drives, and other electronic devices. Sequestration involved members of institute security, the Information Technology department, and an outside forensic computer company. All computer and electronic devices were copied and copies supplied to the laboratory personnel so the affected lab could continue working on research other than that related to the questionable project until the investigation was completed and a decision had been reached. These actions cost an estimated $10,000.00.

#### Investigation

An Investigation Committee was empanelled as described above. Over the course of ten meetings the Committee reviewed all of the questionable lab figures, primary data sources from lab books, electronic data and figures, and e-mail correspondence. The Committee also interviewed the respondent, the complainant, and other members of the laboratory in question. Given the complexity of the case the Investigation Committee was composed of eight individuals who spent well over 100 hours in meetings (∼$78,000) and an estimated 700 hours outside of committee (∼$430,000). Other related costs included transcriptionist and clerical support for photocopying, filing, scheduling, and correspondence (∼$2,500). Moreover, given that the Investigation Committee determined that there was evidence of scientific misconduct, a review of the scientist's other grant applications as well as manuscripts was undertaken. Approximately 50 person-hours were spent reviewing other grants and manuscripts (∼$4,000).

#### Total estimate of costs

We estimate that the direct cost of this case approached $525,000. This includes faculty and witness salaries of about $512,000, clerical support costs of ∼$2,500, and other personnel costs (security, Information Technology, contracted forensics) of ∼$10,000. Other significant costs not factored into the above figure (indirect costs) include deliberation time of senior administrative faculty (CEO, Senior Vice Presidents for Scientific and Translational Research, Executive Vice President, Chair), loss of current grants ($283,000), withdrawal of two pending grant applications (∼$615,000) and one renewal (∼$363,000), the cost to the Institute of maintaining affected pre- and postdocs until other laboratories could be found (∼$40,000), and the cost of maintaining all the records for at least 6 years after the investigation has been completed.

## Closure

The precise prevalence of scientific misconduct is unknown, owing largely to its clandestine nature as well as to the problem of underreporting. Fanelli [2] estimates occurrences between 2% (self) to 14% (others). Other sources cite the risk of misconduct as being less than 1% [4]. The costs associated with institutional investigations are quite significant. We conservatively estimate that if one were to apply our observed costs to all of the allegations of misconduct reported in the United States to the ORI (*n* = 217 cases) in their last reporting year, the direct costs would exceed $110 million. We hope that our work will encourage others to add to our understandings of these costs.

Scientists are people and subject to the frailties of human nature, so we may never be able to totally eliminate scientific misconduct. However, we can prevent those cases of misconduct more related to “omission” of scientific standards rather than commission of misdeeds. How this can be achieved has not yet been determined. Most academic institutions have, like ours, undertaken a number of efforts to increase awareness through education and training, setting forth and enforcing scientific codes of conduct, providing mentorship training, auditing and monitoring procedures, and implementing procedures for reporting and investigating alleged incidents of misconduct. The ultimate effectiveness of these approaches may take time to discern. What is known, however, is that the costs of these proactive activities pale in comparison to the costs of a single case of scientific misconduct.

## Abbreviations

AC: aggregate costs
IC: intangible costs
MC: measurable costs
ORI: Office of Research Integrity
